# Social support and substitute voice acquisition on psychological adjustment among patients after laryngectomy

**DOI:** 10.1007/s00405-016-4310-0

**Published:** 2016-09-29

**Authors:** Kumiko Kotake, Yoshimi Suzukamo, Ichiro Kai, Kazuyo Iwanaga, Aya Takahashi

**Affiliations:** 1 Faculty of Nursing, Nara Medical University, 840 Shijou-cho, Kashihara City, Nara 634-8521 Japan; 2 Physical Medicine and Rehabilitation, Tohoku University School of Medicine, Tohoku, Japan; 3 Graduate School of Medicine and Faculty of Medicine, The University of Tokyo, Tokyo, Japan; 4 Faculty of Medicine, School of Nursing, Fukuoka University, Fukuoka, Japan; 5 Department of Nursing, Saitama Prefectural University, Saitama, Japan

**Keywords:** Social support, Substitute voice acquisition, Psychological adjustment, Laryngectomy

## Abstract

The objective is to clarify whether social support and acquisition of alternative voice enhance the psychological adjustment of laryngectomized patients and which part of the psychological adjustment structure would be influenced by social support. We contacted 1445 patients enrolled in a patient association using mail surveys and 679 patients agreed to participate in the study. The survey items included age, sex, occupation, post-surgery duration, communication method, psychological adjustment (by the Nottingham Adjustment Scale Japanese Laryngectomy Version: NAS-J-L), and the formal support (by Hospital Patient Satisfaction Questionnaire-25: HPSQ-25). Social support and communication methods were added to the three-tier structural model of psychological adjustment shown in our previous study, and a covariance structure analysis was conducted. Formal/informal supports and acquisition of alternative voice influence only the “recognition of oneself as voluntary agent”, the first tier of the three-tier structure of psychological adjustment. The results suggest that social support and acquisition of alternative voice may enhance the recognition of oneself as voluntary agent and promote the psychological adjustment.

## Introduction

In Japan, the number of oral, pharyngeal, and laryngeal (hereinafter perilaryngeal) cancer is reported to be approximately 21,000 [24], a three-fold increase over the last 20 years. In the US, the incidence statistics of cancer site showed that 4 % had head and neck cancer −10,270 laryngeal and 2500 lower pharyngeal in 2004. While the male–female ratio in Japan is 9–1, with more male than female, this ratio in the US has dramatically changed from 15–1 to 5–1. A possible reason for the narrowing gender difference is the increasing smoking population among women [1]. One tenth of perilaryngeal patients undergo partial or total laryngectomy. Since the number of patients with lower pharyngeal cancer has been on the rise, the number of total laryngectomies would not greatly increase in the future.

Many of us typically use voice communication as a necessary component of socialization. Loss of voice suddenly after surgery is considered to cause a significant interference in everyday activity of patients who have undergone laryngectomy (hereinafter laryngectomized patients). They face not only life-time communication difficulties, but also lowered quality of life (QOL) due to physical, psychological, and social adjustment disorders [7–9, 11, 15, 17, 21, 22, 25, 26, 33]. The previous studies reported that some patients showed a depression tendency after the removal of larynx [5, 9, 10, 23, 31] kept themselves in a room and did not communicate with their family members after discharge from hospital [19]. Self-isolation may aggravate depression and lead to lower daily-life satisfaction, lower self-esteem, and role in society [3]. In addition, the mental health of laryngectomized patients was worse than that of depressive patients even before surgery [2]. The psychological factors related to loss of speech are an important issue in which healthcare professionals should pay attention to.

Regarding the psychological adjustment to disability, Dodds et al. [12–14] constructed a structural model by determining seven psychological domains (anxiety/depression, self-esteem, self-efficacy, attitude to disabled people, acceptance of disability, locus of control, and attribution style). In the previous studies for blind people by Dodds et al. [14] and Suzukamo et al. [29, 30], it was suggested that the constructs “self-esteem” and “anxiety/depression” relate to a superordinate concept of “internalized self-worth”, and that the constructs “locus of control” and “self-efficacy” relate to a superordinate concept of “self as an agent”. Based on the model with psychological adjustment for visually impaired people in Japan [28–30], we demonstrated a structure of psychological adjustment for laryngectomized patients [20]. The model consists of a three-tier structure; an increase of recognition of oneself as voluntary agent promotes acceptance of the disability, which further promotes internal value as a human being. The structure is that the constructs “self-esteem” and “anxiety/depression” relate to a superordinate concept of “internal value a human being”, that the constructs “positive affirmation” and “attitude” relate to a superordinate concept of “acceptance of disability”, and that the constructs “locus of control” and “self-efficacy” relate to a superordinate concept of “recognition of oneself as a voluntary agent”.

Social support from people surrounding patients is considered to be essential to the psychological adjustment. However, whether social support influences the psychological adjustment, to what part of its structure, is unknown. There are two types of social support—informal (from family members, friends, or patient associations) and formal (from health care professionals including nurses) [16]. In Japan, a formal support system is not well-established for laryngectomized patients, and cooperation between formal and informal supports is also insufficient [19].

If the alternative voice is not acquired, patients cannot communicate with others smoothly and would have great difficulty in performing their social role. It is reported that approximately 60 % of laryngectomized patients are socially affected due to communication issues [2]. In Japan, 17.4 % of the patients lost their jobs due to voicelessness [18]. Delayed acquisition of alternative voice together with poor psychological adjustment may inhibit their participation in society. However, it is unclear whether psychological adjustment and acquisition of alternative voice are significantly associated.

Laryngectomized patients typically receive speech and voice rehabilitation, such as esophageal speech, electrolarynx (EL), Tapia’s artificial larynx, tracheoesophageal (TE) shunt speech, and speech via computer software. Of these, esophageal speech and TE speech are considered to facilitate psychological/social adjustment, because they enable patients to communicate with relatively close to natural enunciation. However, a great deal of training is required to obtain esophageal speech and keeping the skills is also difficult. The number of patients who use esophageal speech is reported to be 40 % or even less in Japan [18]. TE speech receives attention as a similar method to esophageal speech, but the number of the shunt surgery is not very high and an expensive maintenance cost is also a problem. Given the current situation described above, many patients cannot obtain an alternative voice, so writing and gesturing are the only methods of communication. Armstrong et al. [2] reported that more than 40 % of laryngectomized patients communicate only by writing for the first half year after surgery. If the early acquisition of alternative voice does not succeed, the problems of communication, psychological adjustment, and social role performance may occur.

This study was designed to clarify whether the social support enhances the psychological adjustment of laryngectomized patients and which part in the structure of psychological adjustment the social support influences. We also investigated whether the acquisition of alternative voice influences the psychological adjustment.

## Ethical considerations

Patients were informed that participation was voluntary and that survey respondents would remain anonymous. They were also informed that they would not be disadvantaged in any way if they refused to participate. Patients who signed the consent form or returned a completed questionnaire were considered to have agreed to participate in the survey. The study was reviewed and approved by the Ethics Committee of a University (Approval No. 190045). The study was funded by a grant-in-aid for scientific research from the Ministry of Education, Culture, Sports, Science and Technology (MEXT) in Japan. The patients did not have payment for participating the study.

## Patients and methods

### Study population

We contacted 1445 patients who were enrolled in “B” laryngectomized patient association in 2005. The details of study were explained in written form. The survey form was sent by post. The following information was included: (1) the study protocol was approved by the Ethics Committee of the University “A”, (2) participation in this study is based on voluntary agreement and individuals responding to the questionnaire are not identified, and (3) the study is funded by a grant-in-aid for science research by the Ministry of Education, Culture, Sports, Science and Technology (MEXT) in Japan.

The filled-in questionnaire and consent form were returned only when they agreed to participate in the study. The exclusion criteria were (1) refusal to participate in the study, (2) difficulty with filling out the survey form due to poor physical condition, (3) difficulty with reading due to visual impairment, (4) death, (5) address unknown, and (6) judged by researchers to be difficult to participate for other reasons.

## Study items

### Psychological adjustments

The Japanese version of The Nottingham Adjustment Scale, Laryngectomy (NAS-J-L), for measuring the psychological adjustment of laryngectomized patients, was used [32]. NAS-J-L was a scale modified for laryngectomized patients from NAS; a scale originally developed to measure the psychological adjustment to visual impairments [12, 29].

NAS-J-L consists of 7 subscales and 26 items: (1) the 6 items that represent the “Anxiety-depression of the patient who lost their voice” are hereafter abridged as “Anxiety-depression”. (2) The 2 items that represent the “Self-esteem of the patient” are hereafter abridged as “Self-esteem”. (3) The 3 items that represent the “Self-knowledge into acceptance of disability” are hereafter abridged as “Self-knowledge”: states that even if the voice was lost, the patient does not worry and does not have an unpleasant experience. (4) The 6 items that represent the “Positive affirmation into acceptance of disability” are hereafter abridged as “Positive affirmation”. If the voice is lost, the patient feels positively “It is possible to enjoy life as the person they want to be “, “do things for other people”, “have a significant existence and have a variety of possibilities in life”. (5) The 4 items that represent the “Attitude toward laryngectomized patients” are hereafter abridged as “Attitude”. (6) The 3 items that represent the “Self-efficacy of the patient” are hereafter abridged as “Self-efficacy”, as the patient has a sense that it might be possible to do things for him/herself. (7) The 2 items that represent the “Locus of control of the patient” are hereafter abridged as “Locus of control”.

The higher points in each subscale imply the higher psychological adjustment. The subscales showed sufficient reliability (Cronbach’s α coefficients between 0.60 and 0.91) and construct and criterion-reference validity [32]. The construct validity was shown by a factor analysis, which indicated similar construct as original NAS [32].

### Social support

For informal social support, 20 items of the Medical Outcomes Study Social Support Questionnaire (MOS SSQ) were used. It includes four subscales of emotional/informational, tangible, affectionate, and positive social interaction [27]. For formal social support, 10 items of the Hospital Patient Satisfaction Questionnaire-25 (HPSQ-25) were used. It includes two subscales of technical evaluation and human aspects [6].

### Patient characteristics

Age, sex, and the duration of the post-surgery period were surveyed.

### Communication methods

Communication methods (esophageal speech, electrolarynx, TE speech, writing, and gesturing) and the number of syllables, they were able to produce, were investigated.

Communication methods were injected into each model: a group using esophageal speech and TE speech was coded as 1; a group using writing, gesturing, and electrolarynx only was coded as 0.

## Analysis

To understand the characteristics of the study population, the descriptive analysis was conducted. We then used the model of psychological adjustment of laryngectomized patients developed by our previous study [20]. The structural model consists of a three-tier structure; the “Recognition of oneself as a voluntary agent” (the latent variable for Self-efficacy and Locus of control) promotes “Acceptance of disability” (the latent variable for Positive affirmation and Attitude), which further promotes “Internal value as a human being” (the latent variable for Anxiety/Depression and Self-esteem).

In our preliminary analysis, we found that both informal support and communication methods correlated only to “recognition of oneself as voluntary agent,” but not to “Acceptance of disability” or “Internal value as a human being”. As for patient characteristics, they were not correlated with the three-tier structure variables, and we excluded them from the present analysis.

**Fig. 1:**
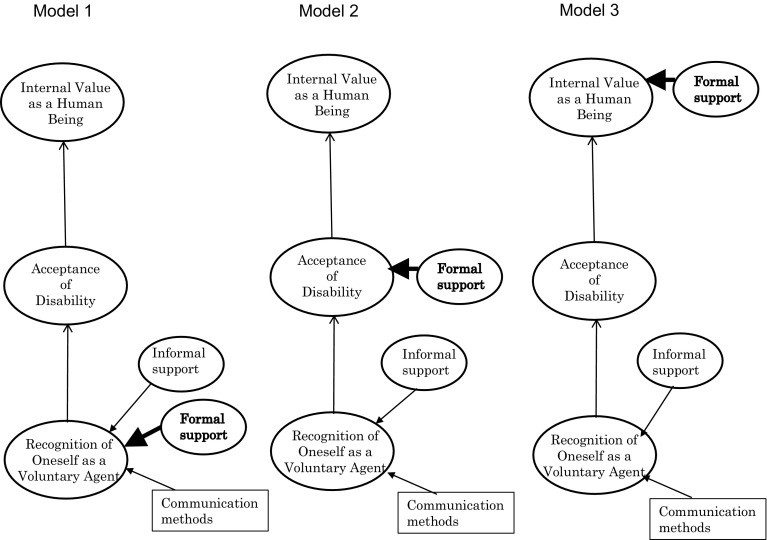
Three hypothetical model

Therefore, we focused whether formal support influenced psychological adjustment in laryngectomized patients this time. In the present study, we established the following three tentative models by adding formal support, and compared them by covariance structure analysis (Fig. 1). Model 1: formal support influences the “recognition of oneself as a voluntary agent”; Model 2: formal support influences the “acceptance of disability”; Model 3: formal support influences the “internal value as a human being”.

## Results

### Participants

Of the 1445 patients to whom the survey form was posted, 131 were excluded: 52 deceased, 17 addresses unknown, 6 hospitalized, 1 difficulty in responding due to old age, and 1 due to frailty. Of the remaining 1314 patients, 679 agreed to participate in the study (response rate 51.7 %).

Baseline characteristics of the study population were published elsewhere [20] and shown in Table 1. The mean age was 70.6 ± 8.3, and they were predominantly male (89.4 %). Of those without occupation, 49.1 % were retired after they reached the retirement age and 26.6 % had lost their jobs due voicelessness. Overall, 30.4 % of the participants had a post-surgery period of 10–20 years. Seventy-four percent of the patients underwent total laryngectomy and 21.8 % esophageal reconstruction.

**Table 1:** Basic characteristics (*N* = 679)

	*N* (%)
Age	
Responded	678 (99.9)
Unknown	1 (0.1)
Mean ± S.D (range)	70.6 ± 8.3 (40–94)
Sex	
Male	604 (89.0)
Female	66 (9.7)
Unknown	9 (1.3)
In the paitents’ household	
1	59 (8.7)
2	340 (50.1)
3	131 (19.3)
4	68 (10.0)
5 or more	63 (9.3)
Unknown	18 (2.7)
Time elapsed after surger (years)	
1 to <3	98 (14.4)
3 to <5	100 (14.7)
5 to <10	181 (26.7)
10 to <20	206 (30.3)
>20	70 (10.3)
Unknown	24 (3.5)
Type of surgery	
Total laryngectomy	502 (74.0)
Esophageal reconstruction	148 (21.8)
Others	9 (1.3)
Unknown	20 (2.9)

Derived from Kotake et al. [20]

### Communication method, psychological adjustment, and social support after laryngectomy

The methods used for communication were published elsewhere [20] and are shown in Table 2. Esophageal speech was used by 465 patients (68.6 %) and TE speech by 17 patients (2.5 %). The number of patients using writing, gesturing, and electrolarynx was 123(18.1 %), 64(9.4 %), and 163 (24.0 %), respectively. More than 65.2 % of the participants were able to produce 5 or more syllables.

**Table 2:** Communication methods after laryngectomy (*N* = 679)

	*N* (%)
Communication methods	
Writing	43 (6.3)
Gesturing	5 (0.7)
Esophageal speech	349 (51.4)
Electrolarynx (EL)	100 (14.7)
Tracheoesophageal (TE) speech	9 (1.3)
Combination of the above	148 (21.8)
Others^a^	5 (0.7)
Unknown	20 (3.0)
No. of syllables possible by esophageal speech	
1 (i.e., “a”)	8 (1.2)
2 (i.e., “a-me”)	5 (0.7)
3 (i.e., “a-ta-ma”)	14 (2.1)
4 (i.e., “o-ha-yo-u”)	20 (2.9)
5 (i.e., “a-ri-ga-to-u”)	442 (65.1)
Unknown	190 (28.0)
Time spent on esophageal speech practice (hrs)	
>2	38 (5.6)
1–2	40 (5.9)
0.5–1	80 (11.8)
<0.5	113 (16.6)
No practice	267 (39.3)
Unknown	141 (20.8)

Derived from Kotake et al. [20]

^a^Email, conversation without voice, observing the shape of the mouth, or Tapia’s artificial larynx

The results of total scores and standard deviation for observed variables under psychological adjustment, communication methods, and informal and formal supports are shown in Table 3. The mean total scores of informal support and formal support were 79.0 ± 19.2 and 68.6 ± 13.7, respectively.

**Table 3:** Scores for informal and formal supports

Observed variables	Total score	
	Mean ± SD (range)	Median
Informal support		
Tangible	83.4 ± 20.2 (0–100)	87.5
Affectionate	79.9 ± 20.2 (0–100)	83.3
Emotional/informational	76.2 ± 20.8 (6.3–100)	78.1
Positive social interaction	76.6 ± 21.0 (0–100)	75.0
Total scores of informal support	79.0 ± 19.2 (5.3–100)	81.6
Formal support		
Technical support	66.8 ± 10.9 (16–83)	66.7
Human support	70.5 ± 18.0 (0–100)	75.0
Total scores of formal support	68.6 ± 13.7 (8–100)	70.8

### Influence of social support and communication methods on the structure of psychological adjustment

#### Model 1

**Fig. 2:**
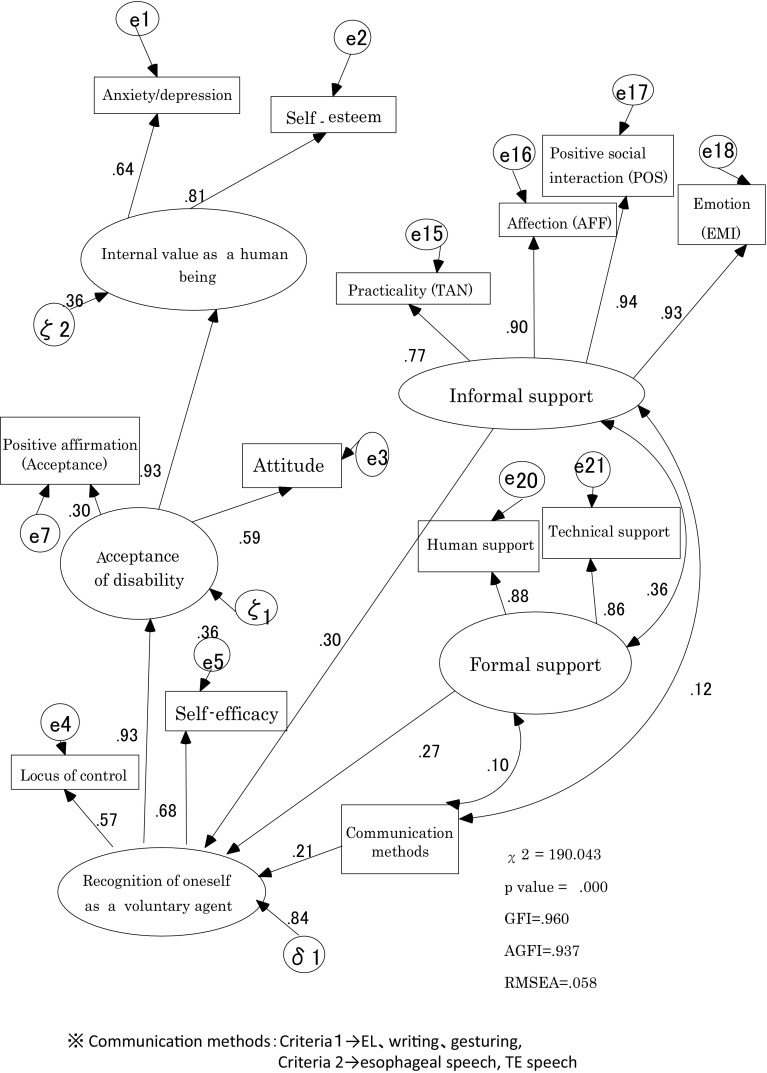
Social support and the influence of communication methods to the structure of psychological adjustment

In the psychological adjustment structure models with the addition of communication methods and informal/formal supports, the model with the highest adjustment is shown in Fig. 2 (GFI = 0.960, AGFI = 0.937, and RMSEA = 0.058). Both informal and formal supports had positive influences on the “recognition of oneself as voluntary agent” (path coefficients 0.30 and 0.27, respectively). Informal and formal supports indicated positive correlation (r = 0.36). Acquisition of alternative voice influenced “recognition of oneself as a voluntary agent” (path coefficient 0.21).

#### Model 2

In Model 2, formal support to the “Acceptance of disability” indicated the path coefficient 0.17 (GFI = 0.957, AGFI = 0.932, and RMSEA = 0.062).

#### Model 3

In Model 3, formal support to the “Internal value as a human being” indicated the path coefficient 0.13 (GFI = 0.955, AGFI = 0.930, and RMSEA = 0.063). Two models were low coefficients.

## Discussion

In this study, we examined the influence of social support and acquisition of alternative voice on the psychological adjustment of laryngectomized patients. Our findings show that social support influences the “recognition of oneself as a voluntary agent” and promotes the psychological adjustment. The acquisition of alternative voice also influences such recognition. A hypothesis of Model 1 of the new model was proved.

## Influence of social support on psychological adjustment

Both informal and formal supports were significantly associated with the “recognition of oneself as a voluntary agent”. It means that the recognition is enhanced by obtaining such support. This recognition is positioned in the first tier of the three-tier structure of psychological adjustment. Our findings show that social support is effective in promoting psychological adjustment of laryngectomized patients through enhancing the recognition.

The “recognition of oneself as a voluntary agent” is defined as the latent factor of self-efficacy and locus of control, representing the concept that one’s actions are determined by way of having a conscious mind which improving motivation. In other words, the sense of control must be created by the individuals themselves. Bandura [4] presented the following four factors to enhance self-efficacy:

1. Achievement of performance (the most important factor. Self-efficacy does not occur spontaneously but does so by the actual experience of success).
2. Substituted experience (observe someone’s successful experience or achievement).
3. Verbal persuasion (verbally explained that one possesses abilities, verbal encouragement).
4. Physiological and emotional uplifting (an uplifting feeling from alcohol, drugs and other factors)

Of the above, verbal persuasion is considered to be obtained through the encouragement of family members and friends and through social support, including explanations from health care professionals. The substituted experience is obtained through social support, such as interactions with laryngectomized patients who have already acquired alternate voice. Bundura may have empirically expressed the influence of social support on self-efficacy. Our findings proved the significant association.

We would like to discuss what type of specific support is anticipated. Considering the contents of the MOS SSQ in using this study, informal support determined and consists of four concepts: (1) availability of information on life after laryngectomy and practical support; (2) mental support; (3) information service; and (4) active social interaction. We consider that for more specific measures based on the above concepts, we may be able to promote or create the following environment, where patients can relax and obtain practical support: advice on the preparation of meals suitable for each patient; support which includes a message that someone is always with them to help; and attendance at patient associations or voice training and involvement in social interaction. Having someone who responds to patients with affection would be effective in promoting the psychological adjustment of the patients. Cooperation and interaction with other patients as well as their family members and friends are greatly needed.

Considering the contents of the HPSQ in using this study, formal support identified in this study includes: (1) relating to the patients with interest and empathy; (2) giving sufficient understanding and consideration to the patients; (3) communicating to soothe their minds; and (4) giving appropriate responses (e.g., nursing, treatment, and respect for patients’ opinion). Based on the above, we may be able to provide opportunities, where patients can have fun and enjoy communicating to one another, such as concerts, Rakugo (Japanese comic story telling), or plenty of conversation with each other. It is also critical to recognize their health condition and provide sufficient care and technical support for their treatment. Furthermore, the establishment of a systematic training course as a follow-up of medical treatment would be necessary, as we should not only depend on voluntary voice training courses in the patient associations.

## Influence of communication methods for psychological adjustment

The “recognition of oneself as a voluntary agent” was enhanced when larnyngectomized patients used communication methods which were close to their own voices, such as esophageal speech and TE speech, in comparison with writing, gesturing, and electrolarynx methods only.

This result suggests that psychological adjustment is enhanced if a patient is capable of esophageal speech and TE speech in their daily life. As previously stated, little formal training has been provided as follow-up treatment in Japan. Therefore, many of the patients who are unable or unwilling to attend the patient associations are not permitted to obtain on alternative voice [19]. To achieve the psychological adjustment of laryngectomized patients, providing a formal support system would be desirable.

As previously stated, one of the important factors in enhancing self-efficacy is the accumulation of “substituted experience”. For the acquisition of an alternative voice, patients are considered to have a “substituted experience” by learning enunciation from other patients. The result, the acquisition of an alternative voice influences the “recognition of oneself as a voluntary agent”, may not only present the fact that the patients acquired an alternative voice, but also be associated with the “substituted experience” in the process of acquiring an alternative voice. The patients who have recently undergone laryngectomy might feel “I may be able to do something. Let’s try it out” through the observation of other patients’ success together with obtaining practical information on how other patients have lived after surgery.

There are limitations to this study. First, the study results cannot yet be applied to patients who have recently had laryngectomy. Since the participants have been enrolled as members of “A” patient association for more than one year, patients in the acute period were not included. Since the patients immediately after laryngectomy tend to need much more social support, the association between the psychological adjustment and social support for the patients in the acute period may be different from that for the patients in the chronic period. In our study, 68.6 % used esophageal speech, and of these, 65.2 % were able to enounce five syllables or more. There is a possibility that the study population included those who have already made progress with their psychological adjustment and have socially well-adapted.

Second, we were unable to determine the causal relation, as this study was not longitudinal. In future studies, we need to observe the psychological change from pre-admission to post-discharge from the hospital and demonstrate how the adjustment is actually structured over time. In addition, it would be useful if we investigate what type of support is needed at each stage together with the efficiency and continuity of the formal support.

## Conclusion

We demonstrated the possibility that formal and informal supports enhance the “recognition of oneself as a voluntary agent” and promote psychological adjustment. The acquisition of an alternative voice also influences the “recognition of oneself as a voluntary agent”. Acquiring esophageal speech and TE speech are superior to writing, gesturing, or EL in view of promoting psychological adjustment.
